# Bioinformatics analysis proposes a possible role for long noncoding RNA MIR17HG in retinoblastoma

**DOI:** 10.1002/cnr2.1933

**Published:** 2024-02-06

**Authors:** Zijin Wang, Xiaotian Liang, Guoguo Yi, Tong Wu, Yuxin Sun, Ziran Zhang, Min Fu

**Affiliations:** ^1^ The Second Clinical Medicine School Southern Medical University Guangzhou Guangdong China; ^2^ Department of Cardiovascular Medicine, Sun Yat‐Sen Memorial Hospital Sun Yat‐Sen University Guangzhou Guangdong China; ^3^ Department of Ophthalmology The Sixth Affiliated Hospital of Sun Yat‐Sen University Guangzhou Guangdong China; ^4^ The First Clinical Medicine School Southern Medical University Guangzhou Guangdong China; ^5^ Department of Ophthalmology, Zhujiang Hospital Southern Medical University Guangzhou Guangdong China

**Keywords:** bioinformatics, competing endogenous RNAs network, long noncoding RNA, MIR17HG, retinoblastoma

## Abstract

**Background:**

Retinoblastoma (RB) is the most common prevalent intraocular malignancy among infants and children, particularly in underdeveloped countries. With advancements in genomics and transcriptomics, noncoding RNAs have been increasingly utilized to investigate the molecular pathology of diverse diseases.

**Aims:**

This study aims to establish the competing endogenous RNAs network associated with RB, analyse the function of mRNAs and lncRNAs, and finds the relevant regulatory network.

**Methods and Results:**

This study establishes a network of competing endogenous RNAs by Spearman correlation analysis and prediction based on RB patients and healthy children. Enrichment analyzes based on Gene Ontology and the Kyoto Encyclopedia of Genes and Genomes are conducted to analyze the potential biological functions of lncRNA and mRNA networks. Weighted gene co‐expression network analysis (WGCNA) is employed to identify gene cluster modules exhibiting the strongest correlation with RB. The results indicate a significant correlation between the lncRNA MIR17HG (*R* = .73, *p* = .02) and the RB phenotype. ceRNA networks reveal downstream miRNAs (hsa‐mir‐425‐5p and hsa‐mir455‐5p) and mRNAs (MDM2, IPO11, and ITGA1) associated with MIR17Hg. As an inhibitor of the p53 signaling pathway, MDM2 can suppress the development of RB.

**Conclusion:**

In conclusion, lncRNAs play a role in RB, and the MIR17HG/hsa‐mir‐425‐5p/MDM2 pathway may contribute to RB development by inhibiting the p53 signaling pathway.

## 1 INTRODUCTION

Retinoblastoma (RB) is the predominant intraocular malignant tumor affecting infants and young children, while its occurrence in adults is rare. There is no difference in race, region, gender, or eye. The mortality rate of RB ranges from approximately 40%–70% in Asia, Africa, and other underdeveloped countries,^1^, ^2^ whereas, it is approximately 3%–5% in Europe, the United States, and other developed countries. The survival rate of children with RB in China is about 63%, which is markedly lower than the survival rates observed in developed countries.^3^ In addition to causing damage to children's vision and ocular health, RB poses a severe threat to their overall well‐being and survival.

In recent decades, advancements in genomics and transcriptomics have unraveled the involvement of noncoding RNA (ncRNA), notably MicroRNAs (miRNAs), and long noncoding RNAs (lncRNAs), in biological and pathological processes, consequently augmenting the intricacy the human genome.^4^, ^5^, ^6^, ^7^ Moreover, this discovery has offered novel avenues for investigating the molecular pathology of various diseases from the primary level.

LncRNAs are composed of more than 200 nucleotides and lack protein‐coding capabilities. However, many ncRNAs regulate important biological processes by regulating transcription and posttranslational modifications.^8^ In subtypes of ncRNAs, microRNAs influence many aspects of metazoan biology primarily by mediating mRNA stability and blocking translation.^9^ LncRNAs have been reported to coordinate chromosome structure and regulate gene transcription during development and human disease.^10^, ^11^ LncRNAs can also act as miRNA decoys or regulate gene expression posttranscriptionally by capturing mRNA in nucleosomes and stress particles. A subset of lncRNAs interferes with translation by disrupting ribosome recruitment and preventing protein phosphorylation.^8^

The competitive endogenous RNA (ceRNA) theory elucidates the intricate interplay between miRNAs, mRNAs, and lncRNAs. Mechanically, lncRNAs can serve as ceRNAs and then bind to miRNA sites, thus modulating the expression of mRNAs and target genes.^12^ While ceRNA network has been constructed in breast cancer,^13^ pancreatic cancer,^14^ and lung cancer,^15^, ^16^ the specific ceRNA networks associated with RB, the pathways marking lncRNA genes, and their role within these complex pathways remain largely unknown.

**FIGURE 1:**
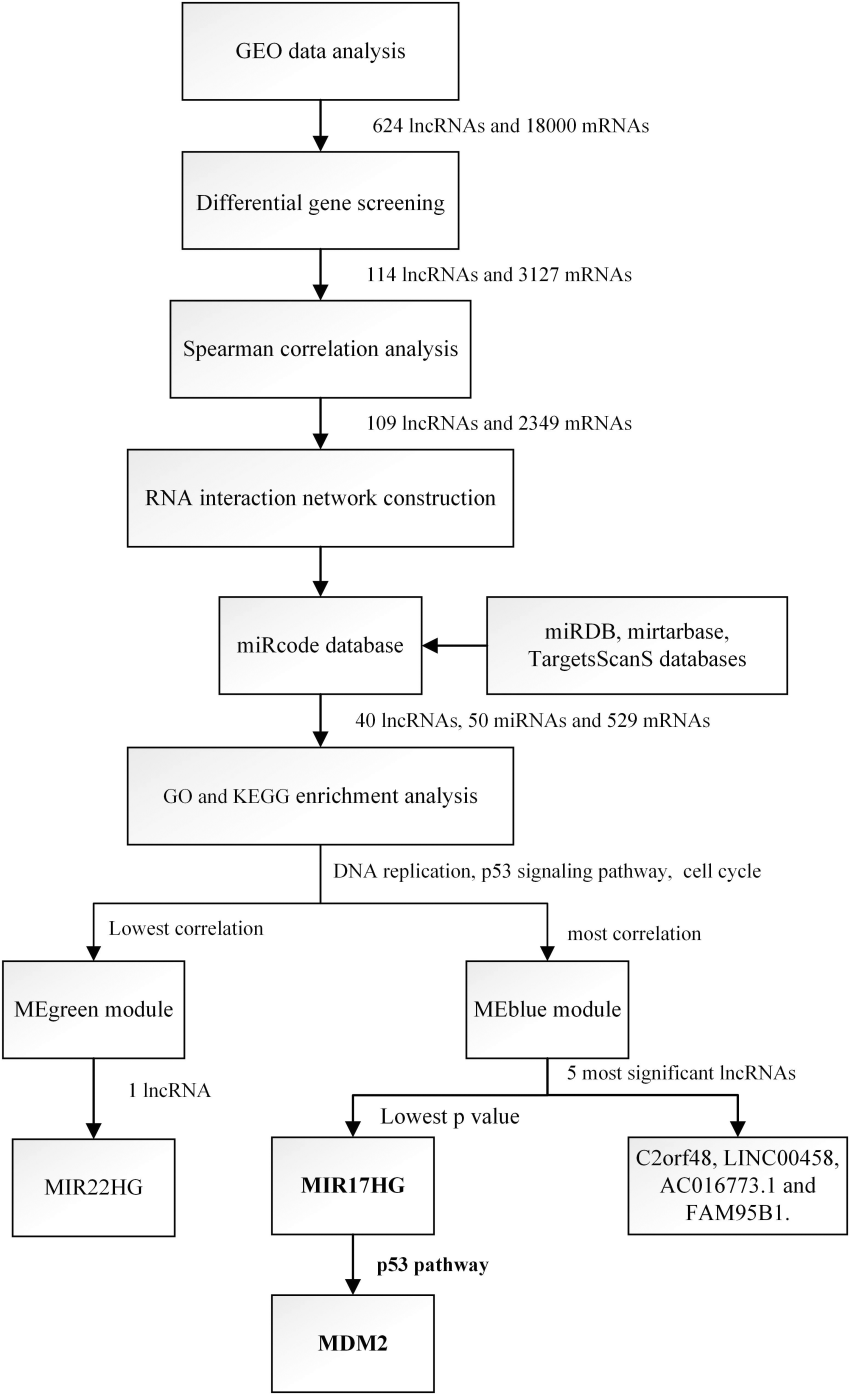
Flowchart of the screening genes and corresponding pathways. This shows the methodology and results of this study to explore the genes and the corresponding pathways. GEO, gene expression omnibus; lncRNA, long noncoding RNA; miRNA, microRNA.

LncRNAs consist of over 200 nucleotides and lack protein‐coding capacity. A variety of lncRNAs are abnormally expressed in RB. TP73‐AS1 and miR‐139‐3p exhibit a negative correlation in RB. Elevated expression of TP73‐AS1 leads to the downregulation of miR‐139‐3p, thereby facilitating the proliferation of Rb cells (excluding migration and invasion). Notably, increased TP73‐AS1 expression demonstrates the ability to decrease miR‐139‐3p expression effectively.^17^ In RB tissue, HOX transcript antisense RNA (HOTAIR) is targeted to inhibit the expression of miR‐613. HOTAIR promotes the expression of c‐met, which is closely related to colorectal cancer, while the upregulation of miR‐613 plays the opposite role. HOTAIR/miR‐613/c‐met signal transduction axis plays an essential role in regulating the survival ability of RB cells.^18^ These findings imply that an imbalance of lncRNAs may influence the progression of RB. Considering the regulatory role of lncRNAs in RB and the mechanism of ceRNA network, this study thereupon probed into the differential expression of lncRNAs and mRNAs between RB patients and normal retina, identify key lncRNAs that could serve as tumor markers for predicting RB, infer the target genes of lncRNAs, construct a ceRNA network, and shed light on the molecular mechanisms underlying RB. These findings contribute to the identification of potential therapeutic targets for RB (Figure 1).

## 2 METHOD

### 2.1 Obtain RB gene expression data

Gene Expression Omnibus (GEO, https://www.ncbi.nlm.nih.gov/geo/) Database is a database for storing chip, second‐generation sequencing, and other high‐throughput sequencing data. The samples utilized in our study were obtained from the GSE125903 dataset sourced from the GEO Database. GSE125903 included three healthy subjects (GSM3584495, GSM3584500, and GSM3584494) and seven RB patients (GSM3584497, GSM3584492, GSM3584499, GSM3584493, GSM3584496, GSM3584491, and GSM3584498). The experimental group consisted of retina samples obtained from children aged 6 months to 6 years, while the control group comprised samples from healthy individuals aged 12, 22, and 5 years. Subsequently, our study designated 10 samples as N1, N2, N3, P1, P2, P3, P4, P5, P6, and P7. The data underwent standardization and were converted into fragments per kilobase million (FPKM) format for differential analysis and co‐expression analysis.

### 2.2 Differential gene analysis and gene annotation

Dataset GSE125903 provides complete data on the differential expression of all genes, including gene ID, fold change, and corrected *p*‐value. We screened out the differentially expressed genes (including coding RNA and ncRNA) in RB by the limma package in R language with the threshold that *p* < .05 and absolute value of log fold change >1. Gencode database (https://www.gencodegenes.org/) provides abundant annotation information for human and mouse genetic material.^19^ We annotated the differentially screened genes using the annotation information from the Gencode database to determine their RNA type.

### 2.3 Construction of co‐expression network

To investigate the relationship between lncRNA and mRNA in the retina, we performed Spearman's correlation analysis to examine the correlation of gene expression and construct the lncRNA‐mRNA co‐expression network using the differentially expressed genes obtained from various samples.^20^ The co‐expression of lncRNA and mRNA in the retina was determined using *p* < .05 and |*R*| >.9 as threshold values.

### 2.4 Construction of ceRNA network

In order to explore the downstream miRNAs and target genes of the 114 differentially expressed lncRNAs, as well as how lncRNAs regulate gene expression in cells, we utilized the miRcode database (http://www.mircode.org/) to predict the downstream miRNAs of the lncRNAs. For the prediction of miRNA target genes, we employed multiple databases, including the miRDB database (http://mirdb.org/), mirtarbase database (http://mirtarbase.cuhk.edu.cn/php/index.php), and TargetsScanS database (http://www.targetscan.org/vert_72/). Furthermore, we searched the predicted downstream miRNAs of lncRNAs in the above database, and selected highly conserved miRNA data to identify their corresponding target genes. The ceRNA network could be constructed, which encompassed the interaction of differential lncRNAs, miRNAs, and target genes.

### 2.5 Gene function analysis

Gene Ontology (GO, http://geneontology.org/) and Kyoto Encyclopedia of Genes and Genomes (KEGG, https://www.kegg.jp/) offer insights into gene enrichment across spatial, molecular, and biological pathway categories. Through the utilization of GO and KEGG analysis, we could understand the functions of genes and the pathways through which they interact. With the aid of David (https://david.ncifcrf.gov/summary.jsp), we performed an analysis of the functional roles of target genes within the predicted ceRNA network, elucidating how lncRNA influences the onset and progression of RB through the regulation of target gene expression. The results of enrichment analysis were screened with *p* < .05 as the threshold.

### 2.6 Weighted gene co‐expression network analysis of coding RNA and ncRNA

The differentially expressed lncRNA, and mRNA profiles were constructed into gene co‐expression networks using weighted gene co‐expression network analysis (WGCNA) package in R language. Due to the complexity and nonlinear nature of the data, the weighted co‐expression relationships among subjects in all datasets were evaluated using Pearson correlation in the adjacency matrix. In this study, the soft threshold parameter is set to *β* = 9 to ensure scale‐free network. The neighbor matrix was utilized to calculate the topological overlap measure (ToM) that represented the degree of overlap in shared neighbors. This approach allowed for the identification of functional modules within the co‐expression network that contained these genes. In addition, the dynamic tree‐cutting algorithm was employed to cluster genes based on ToM, and functional modules consisting of different genes were obtained by merging genes with high similarity.

### 2.7 Cell culture

The Y79 and ARPE‐19 cell lines were purchased from American Type Culture Collection. The Y79 cell line was cultured within the purchased cell culture medium (RPMI‐1640) that contained 10% fetal bovine serum (FBS), and the ARPE‐19 cell line was cultured within the purchased cell culture medium (DMEM F12) that contained 10% FBS. Moreover, the cells were cultured in 5% CO_2_ and 90% humidity at 37°C. The medium would be replaced for every 2 or 3 days.

### 2.8 Quantitative RT‐PCR

Quantitative real time‐polymerase chain reaction (qRT‐PCR) was conducted to measure the expression levels of the selected lncRNA MIR17HG and mRNA murine double minute 2 (MDM2). Total RNA samples were extracted from the Y79 and ARPE‐19 cell lines using TRIzol (Invitrogen, Carlsbad, CA). The relative expression levels of MIR17HG and MDM2 were quantified using ProHS RT‐PCR System (Applied Biosystems, Foster City, USA) according to standard methods of qPCR. MIR17HG (lncRNA): the frontier primer was GTTTTGCCACGTGGATGTGA, the reverse primer was GGCCTCTCCCAAATGGATTGA; MDM2 (mRNA): the frontier primer was GAGCTTGGCTGCTTCTGGG, the reverse primer was GAGCTTGGCTGCTTCTGGG; GAPDH (reference gene): the frontier primer was GGATTTGGTCGTATTGGGCG, the reverse primer was TCCCGTTCTCAGCCATGTAG.

### 2.9 Statistical analysis

Statistical product and service solutions (SPSS) (version 24.0) and R language (version 4.0.3, https://www.r-project.org/) were used for statistical analysis. Spearman correlation analysis was used to identify the correlation between lncRNA and mRNA. *p* < .05 was considered statistically significant.

## 3 RESULTS

### 3.1 Differential gene screening

**FIGURE 2:**
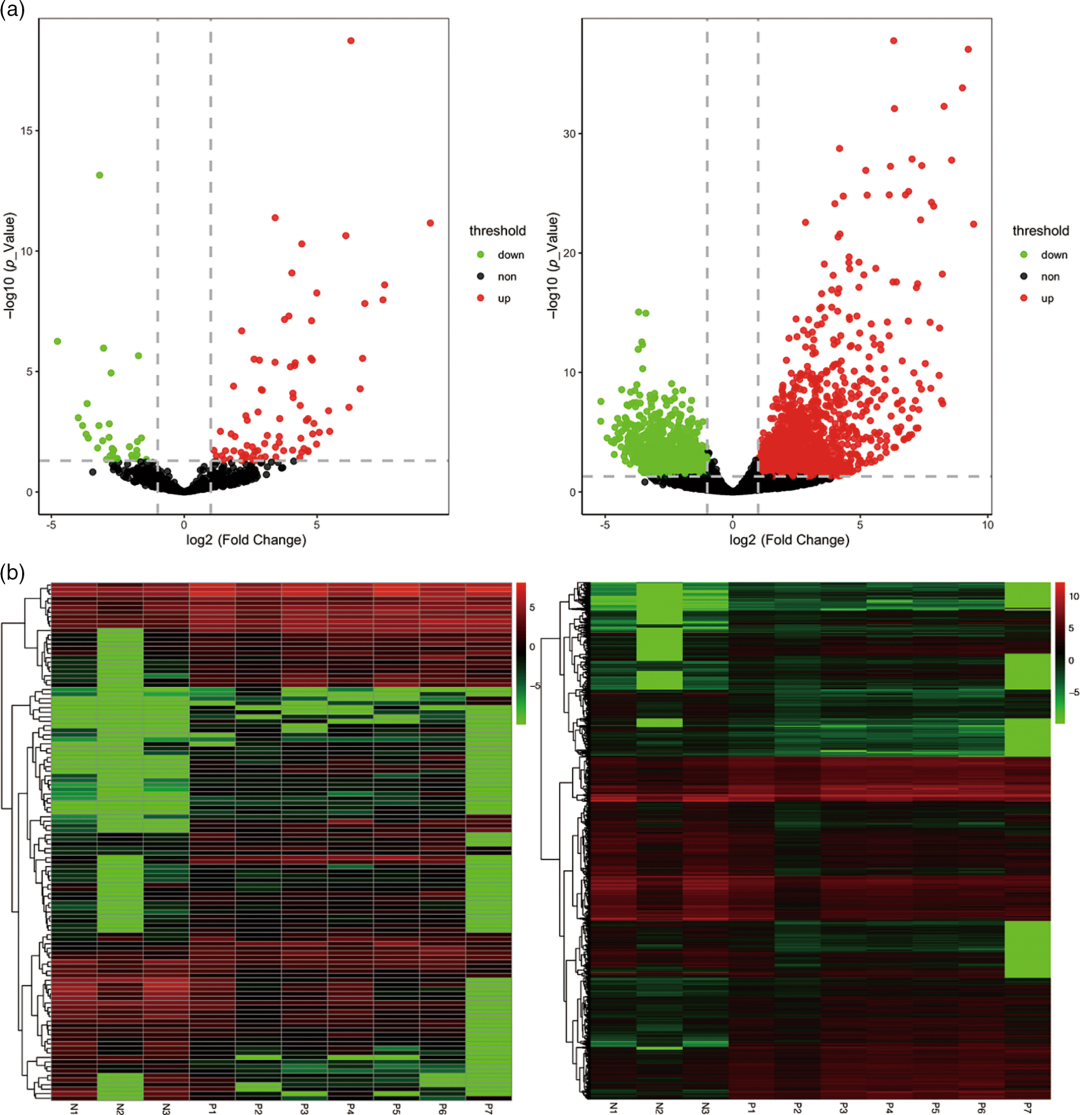
Retinoblastoma gene expression. (A) Volcanic plot of long noncoding RNA (lncRNA) and mRNA. The left pattern is lncRNAs, the right pattern is mRNA. *p* value <.05 and |log2 (fold change)| >1 were considered to be differentially expressed genes. The upregulation and downregulation points are indicated in red and green respectively. It contains 82 upregulated lncRNAs and 32 downregulated lncRNAs. (B) Heat map of the expression levels of lncRNA and mRNA. The original reading was normalized by FPKM format and presented in base 2 logarithm. The left pattern is lncRNAs, the right pattern is mRNA. The expression level was marked by various color, red for high expression and green for low expression. It contains 1626 upregulated mRNAs and 1501 downregulated mRNAs.

Differential gene expression analysis was performed to identify mRNAs and lncRNAs associated with RB, and the identification of lncRNAs and mRNAs was based on the annotation file (Tables S1 and S2). Six hundred twenty four lncRNAs and 18 000 mRNAs were observed in total. A total of 114 lncRNAs showed differential expression, with 82 upregulated and 32 downregulated. In addition, 3127 mRNAs exhibited differential expression, with 1626 upregulated and 1501 downregulated (Figure 2).

### 3.2 Correlation analysis of lncRNAs and mRNAs

To further investigate the correlation between lncRNAs and mRNAs in RB, Spearman correlation analysis was performed between differentially expressed lncRNAs and mRNAs. The co‐expression of lncRNA and mRNA had to meet the thresholds that *p* < .05 and |*R*| >.9, allowing for a strong correlation between the presence of correlation in the final results and the elimination of redundant genes. The resulting co‐expression network comprised 109 lncRNAs and 2349 mRNAs, with the majority showing a positive correlation. Only 12 pairs of lncRNAs and mRNAs exhibited a negative correlation. The analysis revealed that the majority of RB‐related lncRNAs were implicated in carcinogenesis, while the number of suppressor‐type genes was lower.

### 3.3 Prediction of ceRNA network

**FIGURE 3:**
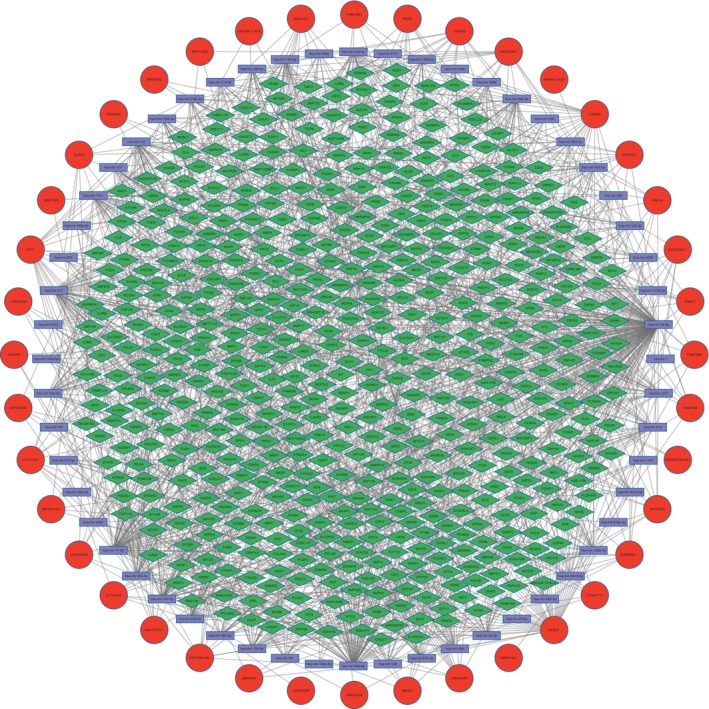
The predicted competitive endogenous RNA (ceRNA) network. Long noncoding RNA (lncRNA) is the red circle, microRNA (miRNA) is the purple rectangle, and target gene is the green diamond. The resulting ceRNA network consisted of 40 lncRNAs, 50 miRNAs, and 529 mRNAs.

To construct an RNA interaction network and obtain the guiding relationships between lncRNAs and mRNAs, we predicted the downstream miRNAs of different lncRNAs with the help of the miRcode database. Subsequently, we identified the potential target genes of the miRNAs using the miRDB, mirtarbase, and TargetsScanS databases. The differentially expressed lncRNAs were connected to the downstream interacting RNAs predicted by the database. Moreover, we retained the lncRNAs and mRNAs that exhibited correlation in the Spearman correlation analysis, thus forming the RNA interaction network. The final predicted ceRNA network obtained comprised 40 lncRNAs, 50 miRNAs, and 529 mRNAs (Figure 3). In the ceRNA network built using Cytoscape, lncRNAs can regulate target genes through various relationships within the network.

### 3.4 Function analysis

**FIGURE 4:**
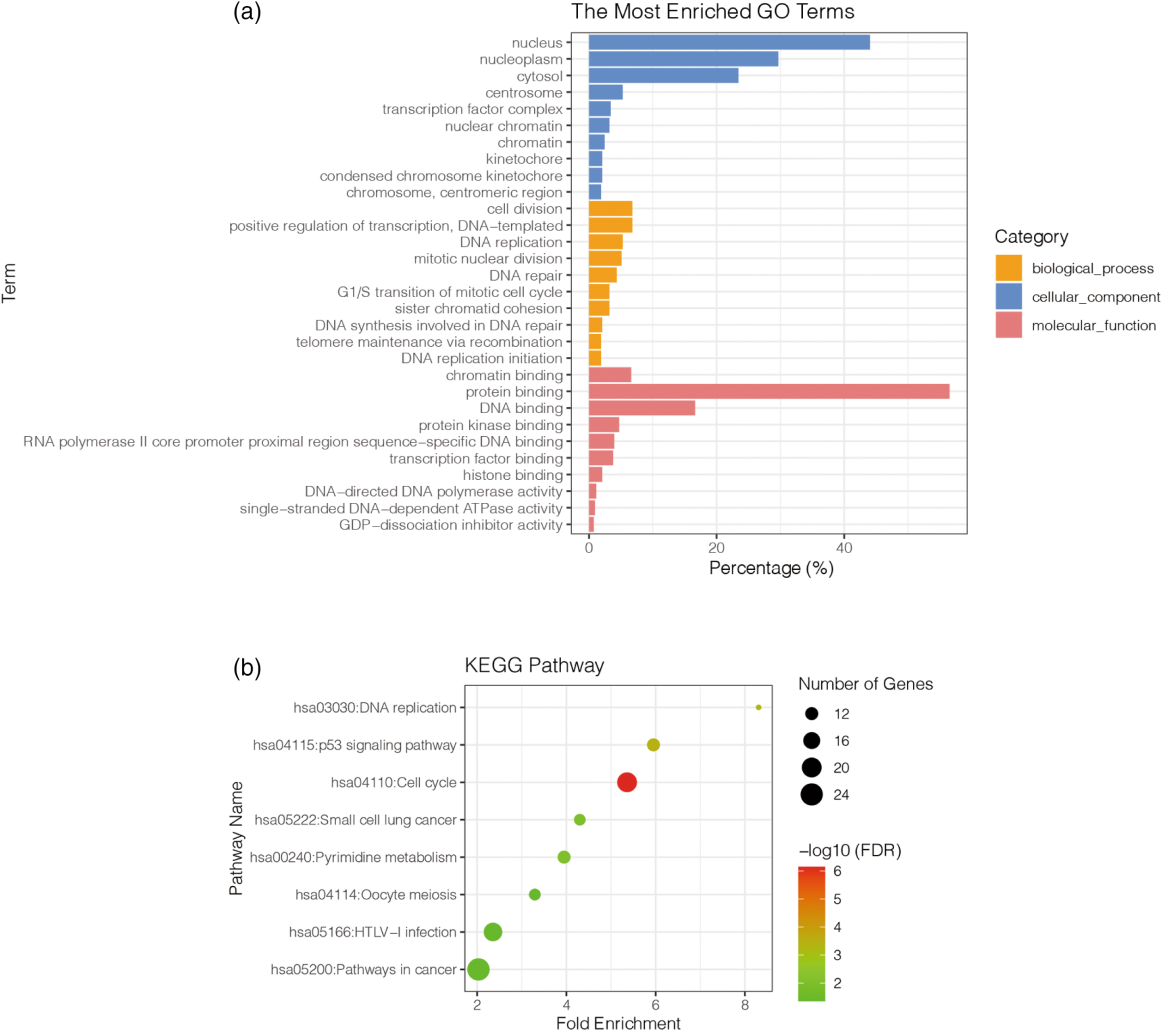
Enrichment analysis of target genes. (A) Enrichment analysis of gene ontology. Cell composition, molecular function, and biological processes are marked with different colors. Abscissa is the ratio of genes enriched in the project to the total genes. All items were screened by false discovery rate (FDR) <0.05. All three items show only the top 10. GDP‐dissociation inhibitor (Guanosine diphosphate dissociation inhibitor) (B) Enrichment analysis of Kyoto encyclopedia of gene and genome (KEGG) pathway. The abscissa is the enrichment degree, the bubble size represents the number of genes enriched in the pathway, and the bubble color represents −log10 (FDR). All pathways were screened by FDR <0.05. DNA replication, the p53 signaling pathway, and the cell cycle exhibited more significant correlations.

The target genes in the predicted ceRNA network were subjected to enrichment analysis in the GO and KEGG databases to determine their functional associations. GO enrichment analysis revealed that the majority of these genes were predominantly localized in the nucleus, nucleoplasm, cytosol, centrosome, and transcription factor complex (Figure 4). The most prevalent biological processes included cell division, positive regulation of transcription, DNA replication, mitotic nuclear division, and DNA repair. In addition, the primary molecular functions associated with these genes encompass chromatin binding, protein binding, DNA binding, protein kinase binding, and sequence‐specific DNA binding in the RNA polymerase II core promoter proximal region.

The results of KEGG analysis revealed enrichment of genes in both upstream and downstream positions of metabolic pathways (Figure 4). Noteworthy pathways include DNA replication, the p53 signaling pathway, the cell cycle, pathways in small cell lung cancer, pyrimidine metabolism, oocyte meiosis, human T‐lymphotropic virus 1 (HTLV‐I) infection, and pathways in cancer. Importantly, DNA replication, the p53 signaling pathway, and the cell cycle are particularly significant pathways that may play a pivotal role in the occurrence and development of RB.

### 3.5 Gene clustering based on WGCNA

**FIGURE 5:**
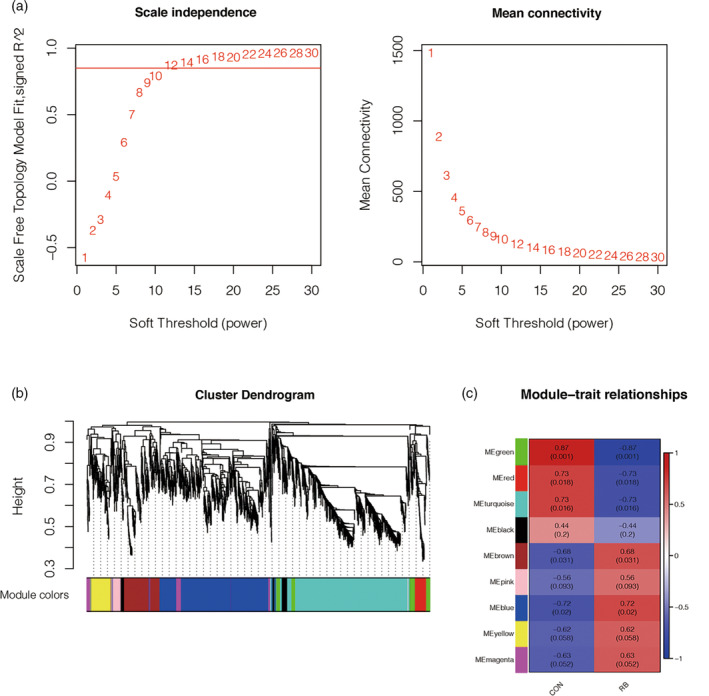
The results of gene clustering. (A) Weighted gene co‐expression network analysis results. Soft threshold is 0.85, *β* is 12, and the average connectivity is shown in the figure. (B) Clustering results of gene modules, and (C) correlation between gene modules and retinoblastoma (RB). The clustering modules are marked with different colors. All genes were grouped into nine modules according to similarity. Of these, the MEblue module had the highest correlation with the RB phenotype (*R* = .72, *p* = .02), while the module with the highest negative correlation with the RB phenotype was the MEgreen module. CON group represented the gene blocks of the healthy control group.

WGCNA was utilized to construct a co‐expression network of differentially expressed lncRNAs and mRNAs in order to identify gene modules associated with RB. The samples were clustered using the average linkage methods and Pearson correlation analysis, and no abnormal outliers were detected. The network topology was analyzed across different soft threshold powers to achieve a balanced scale independence and average connectivity in WGCNA. With an *R* ^2^ > .85 threshold, *β* = 12 was identified as the soft threshold parameter to establish a scale‐free network (Figure 4A). All of the selected genes were clustered using a topological overlap matrix‐based dissimilarity measure based on the dynamic tree cut algorithm, and the genes with more than 25% similarity were combined and divided into nine modules (Figure 5). By analyzing the correlation between gene module and Rb phenotype, we found that MEblue module had the highest correlation with RB phenotype (*R* = .72, *p* = .02). This suggests that some of the genes in the MEblue module are more associated with the prevalence of RB.

**FIGURE 6:**
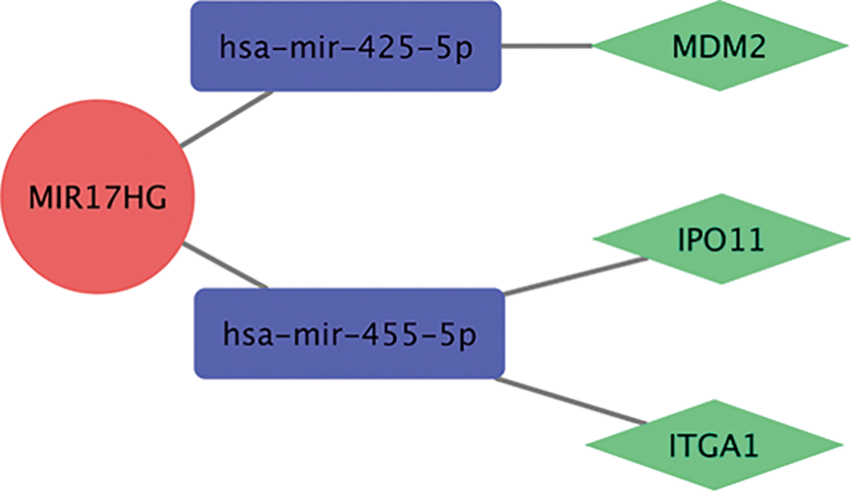
The predicted downstream pathway of MIR17HG. The red circle is lncRNA, the purple rectangle is miRNA, and the green diamond is the target gene. This suggests that MIR17HG regulates MDM2, IPO11, and ITGA1 via hsa‐mir‐452‐5p and hsa‐mir‐455‐5p.

MEblue module contains 977 mRNAs and 28 lncRNAs, and the corresponding relationship between them. This correspondence also exists in the predicted ceRNA network. By comparing and intersecting the two corresponding relationships, we obtained seven lncRNAs along with their downstream miRNAs and mRNAs. The five most significant lncRNAs were MIR17HG, C2orf48, LINC00458, AC016773.1, and FAM95B1. Among them, the MIR17HG has the lowest *p*‐value (logFC = 4.05, *p* = 8.11e−10). We believe that MIR17HG is highly correlated with RB and may play a vital role in the occurrence of RB. Among the three miRNAs and mRNAs associated with MIR17HG, the results of KEGG enrichment analysis revealed the enrichment of MDM2 in the p53 pathway (Figure 6). In addition, the highest negative correlation with the RB phenotype was with the MEgreen module (*p* = .002), and only one lncRNA, MIR22HG (logFC = −3.18, *p* = 2.53e−09), was found when it is intersected with the predicted ceRNA network. Overall, according to the network, MIR22HG regulates FXYD domain‐containing ion transport regulator 3 (FXYD3) through hsa‐mir‐613 and hsa‐mir‐24‐3p, and its downregulation may be associated with tumor formation.

### 3.6 qRT‐PCR validation

**FIGURE 7:**
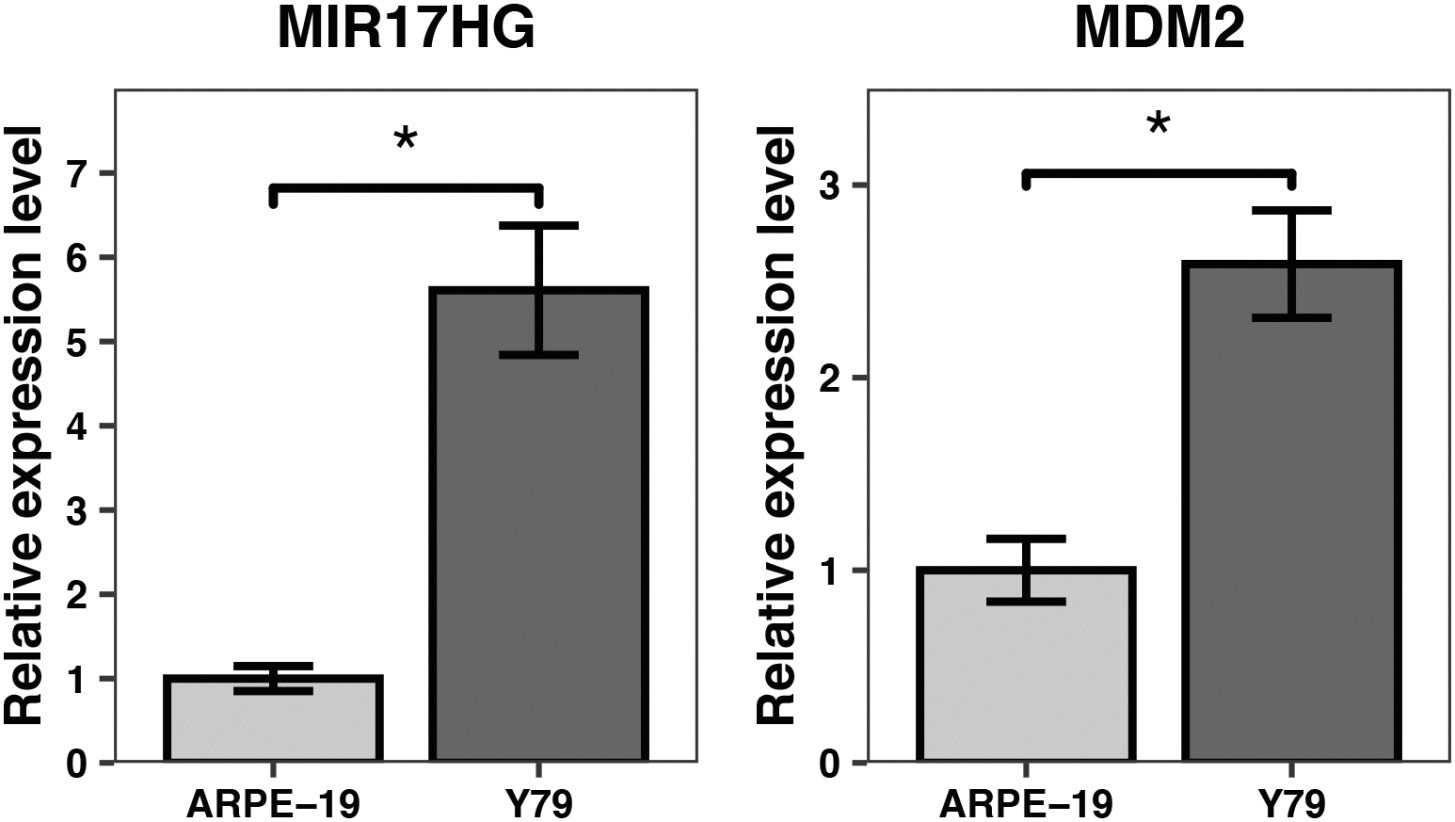
RNA expression of MIR17HG and MDM2. *p*‐values were calculated using two‐sided unpaired Student's *t*‐test. *p < .05. The expression levels of MIR17HG (*p* = .028) and MDM2 (*p* = .016) were significantly higher in retinoblastoma cells compared to control cells, with a significant difference (*p* < .05).

To validate the findings of our bioinformatics analysis, we performed qRT‐PCR to measure the expression levels of MIR17HG and MDM2 in Y79 RB cells and ARPE‐19 control cells. Following a standardized comparison, we observed a significant increase in the expression levels of MIR17HG (*p* = .028) and MDM2 (*p* = .016) in RB cells compared to the control group (Figure 7). The qPCR results validated our hypothesis that MIR17HG as a lncRNA is highly correlated with the onset and progression of RB, and the MDM2 gene is also significantly expressed in miRNAs associated with MIR17HG, a trend that can be seen in the actual RB cells and control cells. Therefore, MIR17G in lncRNAs may affect the onset and progression of RB through the MDM2 gene in miRNAs associated with it. In summary, MIR17HG potentially modulates the MDM2 gene by suppressing the expression of miRNA hsa‐mir‐425‐5p, which in turn relieves p53‐mediated cell cycle inhibition by blocking the p53 signaling pathway, thus playing a role in RB development.

## 4 DISCUSSION

RB is a malignant tumor derived from photoreceptor precursor cells, which is diagnosed within the first few years of life.^21^ Its occurrence is associated with numerous gene mutations, including RB, MYCN, and p53. Genes and gene products constitute a complex network in the process of tumor growth. Analyzing critical nodes and pathways within this network can offer valuable insights for cancer prediction, prevention, and treatment. The advancement of genomics and transcriptomics has highlighted the increasingly crucial role of ncRNA in biological and pathological processes. Exploring ncRNA offers a novel perspective for comprehending the etiology of diseases. According to the ceRNA theory, lncRNAs can regulate other RNA transcripts by competing for and sharing miRNAs. This regulatory network has been confirmed in numerous cancer types.^22^, ^23^, ^24^, ^25^

Previous studies have confirmed that various lncRNAs functioned as ceRNAs to participate in RB pathogenetic process. In this study, by analyzing the samples in GSE125903, we discerned that the lncRNAs and mRNAs differentially expressed in RB and healthy human retina. Subsequently, we obtained the predicted ceRNA network by combination of Spearman correlation analysis and predicting the downstream miRNA of lncRNA and its target genes. Functional analysis of the differentially expressed mRNAs in the aforementioned network unveiled critical molecular mechanisms and metabolic pathways in the retina affected by RB. To improve the accuracy of identifying genes associated with RB, we conducted an intersection analysis between the co‐expression network generated using WGCNA and the predicted ceRNA network. From this analysis, we identified the most significant lncRNAs. Our findings suggest that MIR17HG may regulate the MDM2 gene by downregulating the expression of miRNA hsa‐mir‐425‐5p, potentially alleviating p53‐mediated cell cycle inhibition through blockade of the p53 signaling pathway.

Spearman correlation analysis was conducted to examine the correlation between lncRNA and mRNA expressions. The expression of most mRNAs showed a positive correlation with lncRNA expression, while only 12 mRNAs exhibited a negative correlation. According to the ceRNA network theory, lncRNAs can potentially function as miRNA sponges, thereby alleviating miRNA‐mediated mRNA inhibition and subsequently increasing the expression of target genes. Furthermore, our findings revealed a significant positive correlation between differentially expressed lncRNAs and mRNAs, implying that one possible mechanism by which lncRNAs promote the development of RB is by upregulating the expression of their target genes.

Functional analysis within the predicted ceRNA network revealed significant enrichment of the p53 signaling pathway and cell cycle pathway. Activation of p53 tumor suppressor can lead to cell cycle arrest and induce apoptosis.^26^ On the contrary, the inhibition of p53 pathway may lead to the loss of control of cell cycle. Abnormal cell proliferation plays a crucial role in RB pathogenesis. Studies have shown that p53 pathway is changed in 75% of RB patients, while MDMX and MDM2 are upregulated in 65% and 10% of RB patients, respectively.^27^

MIR17HG was found to promote proliferation, migration, and invasion of RB cells in previous studies.^28^ Among cell lines derived from the human retina, the established ARPE‐19 cell line has similar properties to the endogenous retinal pigment epithelium (RPE) and has therefore been used as a model for analyzing RPE expression profiles.^29^, ^30^, ^31^, ^32^, ^33^, ^34^, ^35^ And in previous studies on RB, most researchers chose ARPE‐19 cells as a control group, and the results of these studies found that there was no significant difference in MIR17HG expression between the two normal retinal cell lines, ARPE‐19 and RPE‐1, so we used the ARPE‐19 cell line as a control group for our experiments.^36^, ^37^, ^38^ On the other hand, the Y79 cell line, as a cell line isolated from a RB patient, is therefore closer to the actual patient's situation at the genetic and molecular levels, so we used the Y79 cell line as the experimental group for our analyzes. Our study reveals that MIR17HG upregulates the expression of MDM2 through mir‐425‐5p in RB. Furthermore, MIR17HG was found to be upregulated in glioma tissues and cell lines, and its downregulation resulted in increased expression of mir‐346/mir‐425‐5p. The direct target of mir‐425‐5p is TAL1, which exhibits a carcinogenic role in glioma cells and can activate the MIR17HG promoter, leading to an upregulation of its expression through a positive feedback loop.^39^ In the current study, MIR17HG was positively correlated with MDM2 expression in RB. As the main negative regulator of p53, MDM2 is a strong inhibitor of apoptosis.^40^ Its encoded protein forms a complex with p53, leading to the inactivation of the p53 gene and the inability to perform its normal tumor‐inhibitory functions, such as apoptosis and cell cycle arrest.^26^ Single nucleotide polymorphisms in the promoter region of MDM2, as well as amplified MDM2 gene, have been observed in various malignant tumors, including breast cancer, lung cancer, esophageal cancer, colon cancer, and various sarcomas,^41^, ^42^ and antagonists of MDM2/MDMX can reactivate the p53 pathway and enhance the apoptosis of RB cells,^43^ while the high expression of MDMX and MDM2 subsequently inhibits the p53 pathway during tumor development.^27^ Mediated by MDM2, E2F protein family of cell cycle related transcription factors, and ADP‐ribosylation factor (ARF) tumor suppressor, RB protein family and p53 have complex interaction.^44^, ^45^, ^46^ The synergistic effect of RB and p53 has been confirmed in bladder urothelial carcinoma, hepatocellular carcinoma, and ovarian cancer.^47^, ^48^, ^49^ MDM2, as an effective regulator of the Rb protein, can degrade RB through two distinct mechanisms: the ubiquitin‐dependent pathway and an independent pathway.^50^ We speculate that MIR17HG may upregulate the expression of MDM2 by competitively binding mir‐425‐5p and inhibit the anticancer effect of RB protein and p53 tumor suppressor gene. This process is believed to be involved in the occurrence and development of RB.

Furthermore, MDM2 has been shown to possess carcinogenic effects independent of its role as a p53 antagonist. MDM2 stabilizes the E2F1 protein through ubiquitination, and this protein is involved in various cellular processes, such as cell cycle progression, apoptosis, and DNA repair.^51^ As a ubiquitin E3 ligase that facilitates the degradation of p53 through the proteasome, MDM2 can promote the ubiquitination of p21 (WAF1/CIP1), resulting in its degradation and contributing to carcinogenesis.^52^ Apart from its regulation of MDM2, MIR17HG exhibits independent carcinogenic properties. MIR17HG can promote the progression of colorectal cancer by competitively reducing the expression of mir‐375.^53^ In addition, MIR17HG can enhance the expression of mir‐18a and mir‐19a, suppress the expression of Smad2, and upregulate Wnt/β‐Catenin signaling, thereby facilitating the metastasis of gastric cancer cells.^54^ In conclusion, the regulation of the MDM2 gene by MIR17HG, which leads to the downregulation of miRNA hsa‐mir‐425‐5p levels during RB development, can alleviate p53‐mediated inhibition of the cell cycle by blocking the p53 signaling pathway. This pathway is of significant importance and holds promise for future RB treatments.

Another mRNA regulated by MIR17HG in our findings is IPO11. The activation of the Wnt signaling pathway relies on the stabilization and nuclear translocation of β‐Catenin protein to regulate context‐specific transcription processes. In contrast, importin‐11 (ipo11), as a nuclear import protein, can transport goods from the cytoplasm to the nucleus. The downregulation of nuclear β‐Catenin protein levels and the subsequent decrease in target gene activation observed in IPO11‐deficient cells (−/−) indicate that IPO11 facilitates the nuclear import of β‐Catenin, while the knockout of IPO11 leads to reduced colony formation in colorectal cancer cell lines.^55^ Moreover, ipo11 is also highly expressed in bladder cancer.^56^ Therefore, IPO11 may be involved in inhibiting the genesis and development of RB under the regulatory influence of MIR17HG. However, this hypothesis needs further experimental validation.

As mentioned previously, in addition to MIR17HG, we identified several other lncRNAs in the MEblue module that have been shown to have a strong correlation with RB: C2orf48, LINC00458, and AC016773.1, and all of these lncRNAs have been shown to play a role in multiple tumorigenesis. RRM2‐C2orf48 chimeric transcript is highly expressed in human nasopharyngeal carcinoma (NPC), and it is speculated to be an effective predictor of NPC metastasis potential.^57^ Moreover, C2orf48 has been identified in the RNA network analysis of endometrial carcinoma,^58^, ^59^ non‐small cell lung cancer,^60^ tongue squamous cell carcinoma,^61^ and hepatocellular carcinoma.^62^ E2F family is a vital transcription activator that regulates cell cycle; it modulates protein expression and DNA synthesis essential for the G1‐S phase transition, consequently influencing cell differentiation, aging, and apoptosis.^44^ C2orf48 modulates E2F1 expression through MIR23B/MIR10A, as predicted in our study. This suggests that C2orf48 might contribute to the progression of RB by regulating E2F1. MIR17HG gene serves as the host gene for the MIR17‐92 cluster, which encompasses miRNA miR‐17. miR‐17 regulates the gene and protein expression of RB by targeting RB mRNA‐3′UTR, thus regulating the proliferation of vascular smooth muscle cells.^63^ It is speculated that miR‐17 and LINC00458 may be related to the vascular changes of RB. AC016773.1 forms a ceRNA network comprising 93 lncRNAs, 9 miRNAs, and 9 mRNAs in laryngeal squamous cell carcinoma (LSCC) and holds promise as a crucial biomarker for LSCC prognosis.^64^ Nevertheless, the current literature lacks sufficient studies examining the association between RB and the aforementioned lncRNAs. Hence, it is advisable to conduct additional analyzes and experiments in the future to validate the relationship between these lncRNAs, their associated pathways, and the onset and progression of RB.

Nonetheless, this study has several limitations. First, our sample size consisted of only seven RB samples and three healthy retina samples. Therefore, to ensure more reliable and precise results and conclusions, further cross analysis with a larger sample cohort is imperative. Second, we employed qRT‐PCR exclusively to validate the expression levels of lncRNAs and MDM2 within the ceRNA networks. To delve deeper into the regulatory dynamics among MIR17HG, mir‐425‐5p, and MDM2, we relied solely on bioinformatic analysis methods to explore these relationships. In subsequent experiments, we intend to supplement our findings by employing the qPCR method to confirm mir‐425‐5p expression levels in RB. Furthermore, it is crucial to emphasize that our conjecture regarding the upregulation of MIR17HG and its potential carcinogenic impact on MDM2 is grounded solely in transcriptional expression levels and correlations. To derive more profound and reliable conclusions, further investigation is warranted. In forthcoming studies, we plan to amalgamate the RB‐related genes identified in this research for additional experimental scrutiny, with the aim of uncovering additional RB‐related pathways and making substantial contributions to the understanding of RB mechanisms.

In conclusion, this study validated that MIR17HG is altered in RB. Moreover, our pathway screening revealed that MIR17HG potentially regulates the MDM2 gene by downregulating the level of miRNA hsa‐mir‐425‐5p, thereby mitigating the cell cycle inhibition mediated by p53 through blockade of the p53 signaling pathway. These findings offer a novel avenue for the identification of therapeutic targets related to RB genes in future research.

## AUTHOR CONTRIBUTIONS

*Conceptualization*: Xiaotian Liang, Guoguo Yi, and Min Fu; *Methodology*: Zijin Wang and Xiaotian Liang; *Formal analysis*: Zijin Wang and Xiaotian Liang; *Writing—original draft*: Zijin Wang and Xiaotian Liang; *Writing—review and editing*: Zijin Wang, Wu Tong, Yuxin Sun, and Ziran Zhang; *Visualization*: Zijin Wang and Xiaotian Liang; *Software*: Zijin Wang and Xiaotian Liang; *Supervision*: Guoguo Yi and Min Fu. All authors approved the final manuscript as submitted and agree to be accountable for all aspects of the work.

## FUNDING INFORMATION

This research was supported by the Natural Sciences Foundation of Hunan Province (2017JJ2200), the Scientific Research Fund of Hunan Provincial Education Department (19A355, 18C1147).

## CONFLICT OF INTEREST STATEMENT

The authors have stated explicitly that there are no conflicts of interest in connection with this article.

## ETHICS STATEMENT

There are no human subjects in this article and informed consent is not applicable.

## Supporting information


**Data S1:** Supporting Information.

## Data Availability

The data that support the findings of this study are available from the corresponding author upon reasonable request.
