# Supplementary material for: Bioinformatics analysis proposes a possible role for long noncoding RNA MIR17HG in retinoblastoma
Source: Cancer Rep (Hoboken). 2024 Feb 6;7(2):e1933. doi: 10.1002/cnr2.1933 (PMC10864729; doi:10.1002/cnr2.1933)
Supplement: Supplementary file 1 — Data S1: Supporting Information. [file CNR2-7-e1933-s001.docx]

**Supplementary Online Content**

**eTable 1. Differentially expressed lncRNA**

**eTable 2. Differentially expressed mRNA**

**eTable 1.** Differentially expressed lncRNA

| lncRNA^a)^ | Biotype | log FC | P. Value | Change |
| --- | --- | --- | --- | --- |
| MNX1-AS1 | lincRNA | 9.263172123 | 6.86046E-12 | Up |
| C2orf48 | lincRNA | 7.541301534 | 2.53031E-09 | Up |
| LINC00458 | lincRNA | 7.478381889 | 1.05799E-08 | Up |
| U95743.1 | lincRNA | 6.795454172 | 1.50511E-08 | Up |
| SHANK2-AS2 | antisense | 6.712941113 | 2.84763E-06 | Up |
| KCNH1-IT1 | sense_intronic | 6.61798464 | 5.20801E-05 | Up |
| AC092198.1 | lincRNA | 6.273706473 | 1.85454E-19 | Up |
| HOXC-AS1 | antisense | 6.200666046 | 0.000304199 | Up |
| C17orf102 | bidirectional_promoter_lncRNA | 6.081040611 | 2.28748E-11 | Up |
| LINC00469 | lincRNA | 5.474574793 | 0.003053425 | Up |
| LINC00844 | lincRNA | -4.771603431 | 5.58628E-07 | Down |
| FAM138E | lincRNA | -3.98973325 | 0.000827989 | Down |
| AP000997.1 | lincRNA | -3.824719342 | 0.001753702 | Down |
| FEZF1-AS1 | antisense | -3.691241155 | 0.003988662 | Down |
| AP000997.2 | lincRNA | -3.655449015 | 0.000214449 | Down |
| AC067969.2 | antisense | -3.609763063 | 0.005796003 | Down |
| LINC00290 | lincRNA | -3.258229667 | 0.01482495 | Down |
| WWC2-AS1 | antisense | -3.210597737 | 0.001752245 | Down |
| MIR22HG | lincRNA | -3.186011844 | 7.04462E-14 | Down |
| FAM138B | lincRNA | -3.069066577 | 0.007490536 | Down |

^a)^ Only top 10 of up-regulation and down-regularion are shown.

**eTable 2.** Differentially expressed mRNA

| mRNA^a)^ | Biotype | log FC | P.Value | Change |
| --- | --- | --- | --- | --- |
| DEPDC1 | protein_coding | 9.445151963 | 3.83135E-23 | Up |
| CKAP2L | protein_coding | 9.23314843 | 8.79968E-38 | Up |
| NDC80 | protein_coding | 9.006471316 | 1.48738E-34 | Up |
| IGFBPL1 | protein_coding | 8.58349373 | 1.6688E-28 | Up |
| KIF20A | protein_coding | 8.284487863 | 5.23606E-33 | Up |
| GATA4 | protein_coding | 8.234526313 | 4.08131E-08 | Up |
| NEIL3 | protein_coding | 8.216247219 | 5.81863E-19 | Up |
| SHOX2 | protein_coding | 8.185327142 | 2.26016E-08 | Up |
| MNX1 | protein_coding | 8.109894622 | 1.91098E-14 | Up |
| SP9 | protein_coding | 8.093893651 | 1.752E-10 | Up |
| PPEF2 | protein_coding | -5.176531413 | 1.21149E-06 | Down |
| RRH | protein_coding | -5.169419587 | 2.67058E-08 | Down |
| WFDC10B | protein_coding | -4.923376898 | 3.04242E-05 | Down |
| ESRRB | protein_coding | -4.757591109 | 6.09942E-05 | Down |
| GNAT1 | protein_coding | -4.653239313 | 7.71043E-05 | Down |
| RHO | protein_coding | -4.64574676 | 0.000736106 | Down |
| RGR | protein_coding | -4.621797827 | 3.94567E-06 | Down |
| CHRNE | protein_coding | -4.61520792 | 4.339E-05 | Down |
| CA14 | protein_coding | -4.512605111 | 7.0006E-07 | Down |
| CLUL1 | protein_coding | -4.502344201 | 3.95344E-05 | Down |

^a)^ Only top 10 of up-regulation and down- regulation are shown.
